# *Cish* knockout mice exhibit similar outcomes to malaria infection despite altered hematopoietic responses

**DOI:** 10.3389/fmicb.2023.1288876

**Published:** 2023-11-02

**Authors:** Asha L. Lakkavaram, Saeed Maymand, Wasan Naser, Alister C. Ward, Tania F. de Koning-Ward

**Affiliations:** ^1^School of Medicine, Deakin University, Waurn Ponds, VIC, Australia; ^2^College of Science, University of Baghdad, Baghdad, Iraq; ^3^Institute for Mental and Physical Health and Clinical Translation, Geelong, VIC, Australia

**Keywords:** *Plasmodium*, CISH, erythropoiesis, hematopoiesis, anemia, cerebral malaria

## Abstract

The Cytokine-inducible Src homology 2 domain-containing (CISH) protein is a negative feedback regulator induced by cytokines that play key roles in immunity and erythropoiesis. Single nucleotide polymorphisms (SNPs) in the human *CISH* gene have been associated with increased susceptibility to severe malaria disease. To directly assess how CISH might influence outcomes in the BALB/c model of malaria anemia, CISH knockout (*Cish*^−/−^) mice on this background were infected with *Plasmodium berghei* and their hematopoietic responses, cytokine production and ability to succumb to severe malaria disease evaluated. Despite basal erythrocytic disruption, upon *P. berghei* infection, the *Cish ^−/−^* mice were better able to maintain peripheral blood cell counts, hemoglobin levels and a steady-state pattern of erythroid differentiation compared to wild-type (*Cish*^+/+^) mice. Ablation of CISH, however, did not influence the outcome of acute malaria infections in either the BALB/c model or the alternative C57BL/6 model of experimental cerebral malaria, with the kinetics of infection, parasite load, weight loss and cytokine responses being similar between *Cish*^+/+^ and *Cish^−/−^* mice, and both genotypes succumbed to experimental cerebral malaria within a comparable timeframe.

## Introduction

Malaria is a globally important disease caused by parasites from the genus *Plasmodium*. Approximately 247 million new cases of malaria occur each year, with the majority of the estimated 619,000 deaths in 2021 occurring in sub-Saharan Africa and from infection with *P. falciparum* (World Health Organization, 2022). The most common syndromes associated with severe or complicated malaria include severe malaria anemia, cerebral malaria (CM) and respiratory distress, either singly or in combination (Moxon et al., 2020). The causes of severe malaria anemia are multi-factorial and include the destruction of parasitized red blood cells (pRBCs), clearance of uninfected RBC, suppression of erythropoiesis and dyserythropoiesis [reviewed in Lamikanra et al. (2007) and Moxon et al. (2020)]. CM is a severe neurological complication, leading to coma and death, and arises from several pathophysiological mechanisms, including sequestration and microvascular obstruction of pRBC (MacPherson et al., 1985; Pongponratn et al., 2003), liberation of pro-inflammatory mediators, dysregulation of coagulation pathways (van der Heyde et al., 2006; Francischetti et al., 2008; Moxon et al., 2009) and disruption of the blood brain barrier (Beare et al., 2009; Dorovini-Zis et al., 2011). Respiratory distress stems from metabolic acidosis leading to respiratory compensation and tissue hypoxia but can also result from damage to the lung due to the infiltration of leukocytes, microhemorrhages and pulmonary edema (Taylor et al., 2012).

The risk of succumbing to severe disease upon *Plasmodium* infection is dependent on the age and immune status of the infected individual. Most malaria deaths in endemic regions of Africa are seen in young children but in regions such as Asia, where transmission is lower, severe disease occurs in older children and adults. Environmental, socioeconomic and host genetic factors also contribute to the varied responses to infection (Weatherall and Clegg, 2002; Marquet, 2018). For example, mutations and polymorphisms in a variety of human genes, such as those mediating glucose-6-phosphate dehydrogenase deficiency, sickle cell trait and other hemoglobinopathies, have been associated with malaria resistance through linkage analysis and association studies (Driss et al., 2011; Marquet, 2018). However, dissecting human genetic susceptibility to malaria has been challenging because of the heterogeneity in the clinical presentations of malaria. Moreover, selection pressures on human genes might differ between populations and rather than common variants in a small number of genes, rare mutations in multiple genes may be involved. As a consequence, inconsistent findings between studies are frequently observed (Marquet, 2018).

In more recent times, genome-wide association studies (GWAS) and genotyping tools have identified genetic polymorphisms associated with altered susceptibility to *P. falciparum* malaria, which may pave the way to fully understanding the pathogenesis of severe disease (Driss et al., 2011; Marquet, 2018). In one such study, five single nucleotide polymorphisms (SNPs) in the gene encoding the human cytokine-inducible Src homology 2 domain-containing (CISH) protein were associated in African and Asian cohorts with increased susceptibility to severe manifestations of malaria infections, as well as to bacteremia and tuberculosis (Khor et al., 2010). The overall risk of contracting one of these infectious diseases was increased by >18% for people harboring the variant *CISH* alleles. However, SNP -292 accounted for most of the association and was shown to correlate with reduced induction of *CISH* expression by IL-2 *ex vivo* (Khor et al., 2010). Subsequent studies have also revealed associations between *CISH* polymorphisms and increased susceptibility to tuberculosis or chronic hepatitis B across Asian and African populations (Wang and Wang, 2010; Ji et al., 2014; Sun et al., 2014; Zhao et al., 2014; Naderi et al., 2016).

CISH is a member of the suppressor of cytokine signaling (SOCS) family of negative feedback regulators (Trengove and Ward, 2013; Sobah et al., 2021). The *CISH* gene is typically induced by cytokines that activate the transcription factor Signal transducer and activator of transcription 5 (STAT5), including erythropoietin (EPO), granulocyte-macrophage colony-stimulating factor (GM-CSF), interleukin (IL)-2, IL-3, IL-15, growth hormone and prolactin (Yoshimura et al., 1995; Matsumoto et al., 1997; Adams et al., 1998; Helman et al., 1998; Hunter et al., 2004). CISH is also induced by IL-4 signaling via STAT6 (Liu et al., 2009) and T cell receptor signaling in a STAT-independent manner (Aman et al., 1999; Li et al., 2000; Yoshimura et al., 2007).

CISH plays a critical role in immunity and has been shown to regulate the development and homeostasis of T cells, NK cells, dendritic cells and myeloid cells (Yang et al., 2013; Palmer et al., 2015; Delconte et al., 2016; Louis et al., 2020; Naser et al., 2023), and also plays a role in the regulation of erythropoiesis (Maymand et al., 2023). We hypothesized that ablation of *CISH* could increase susceptibility to severe malaria anemia and cerebral malaria through its impacts on erythropoiesis and/or immunity, possibly via altered inflammatory cytokine responses. To examine this hypothesis, wildtype (*Cish^+/+^*) and CISH knockout (*Cish^−/−^*) mice were compared in mouse models of malaria anemia (Craig et al., 2012) or experimental cerebral malaria (ECM) (de Oca et al., 2013) resulting from elevated pro-inflammatory immune responses (Hanum et al., 2003). This allowed examination of the impact of CISH ablation on hematopoiesis, inflammatory cytokine levels and susceptibility of mice to severe disease.

## Methods

### Mice and ethics approval

*Cish^+/+^* and knockout *Cish^−/−^* mice (Naser et al., 2022) on either a BALB/c or C57BL/6 background were backcrossed 8 times followed by an intercross to generate *Cish^+/+^* and *Cish^−/−^* founders that were maintained as separate lines. Mice were fed a standard rodent diet (chow) and housed under controlled conditions at 21 ° C with a 12:12 h light:dark cycle. Female mice between 6–10 weeks of age were used for the experiments unless otherwise stated. All experiments were approved by the Deakin University Animal Welfare Committee (Projects G37/2013 and G09/2017) and performed in accordance with National Health and Medical Research Council recommendations in the ‘Australian code for the care and use of animals for scientific purposes’.

### *Plasmodium berghei* infection studies

Mice were infected with wildtype *P. berghei* ANKA (10^6^ parasitized RBCs unless otherwise stated) by intraperitoneal injection and relative parasitemia determined by visualization of Giemsa-stained blood smears and counting a minimum of 1,000 RBC. For the malaria anemia studies, BALB/c mice were used. For the experimental cerebral malaria studies, C57BL/6 mice, which are susceptible to cerebral malaria, were used. Mice were monitored for cerebral malaria symptoms, including ataxia, limb paralysis, presence of seizures and inability to self-right and mice were humanely culled when at they displayed at least three of these symptoms.

### Mouse blood analysis

Blood was collected from the tail of mice with minivets (Sarstedt™) and analyzed with a hematology analyzer (SCIL) according to the manufacturer’s instructions.

### Harvesting of mouse bone marrow and spleen cells

Bone marrow was extracted from the tibias and femurs using a 26-gauge needle and 1 mL RPMI 1640 media (Life Technologies). The entire spleen was placed in 5 mL media and passed through a 40 μm nylon mesh cell strainer (Interpath) to isolate single splenocytes. The cells were then centrifuged at 1,000 × *g* prior to resuspension in the appropriate buffer.

### Antibody staining and flow cytometric analysis

Bone marrow cells or splenocytes (1 × 10^6^ cells/50 μL) in PBS buffer containing 1% (w/v) bovine serum albumin (BSA) were incubated for 15 min on ice with anti-mouse CD16/CD32 (Fcγ III/II receptor; 2.5 μg/10^6^ cells). Samples were then stained with the following antibodies, alone or in different combinations on ice for 20 to 30 min in the dark: PE-conjugated anti-mouse Ter-119 (0.5 μg/10^6^ cells), APC-conjugated anti-mouse CD44 (clone IM7; 0.2 μg/10^6^ cells), APC-Cy7-conjugated anti-rat CD11b (M1/70; 0.1 μg/10^6^ cells), FITC-conjugated anti-mouse Gr-1 (clone RB6-8C5; 0.1 μg/10^6^ cells), PE-conjugated anti-mouse Ly6G (clone 1A8; 0.1 μg/10^6^ cells) and/or BV421-conjugated anti-mouse CD11c (clone N418; 0.2 μg/10^6^ cells). An unstained population of cells were used as a negative control. Cells were pelleted at 1000 × *g* and then washed in PBS containing 1% (w/v) BSA before finally resuspending in 0.2 mL PBS containing 1% (w/v) BSA. Cells were subsequently incubated with 7-AAD (0.1 μg1 × 10^6^) cells for 10 min prior to FACS analysis. Between 10,000–10,000,000 stained cells were analyzed by flow cytometry with a BD FACS CANTO II flow cytometer (BD Biosciences). Gating on FSC/SSC properties was used to remove cell debris and noise, single cells were gated based on FSC area to height ratio and live cells were gated using 7-AAD. Compensation and further analyses were performed using FlowJo v.10.0.6 (Tree Star).

### Methyl cellulose colony assay

This assay was performed as previously described (Lakkavaram et al., 2020). Briefly, bone marrow and spleen cells (5 × 10^5^ cells/100 μL) in Iscove’s modified Dulbecco’s medium (IMDM) (Sigma) containing 2% (v/v) FBS were gently mixed with 1 mL Mouse Methyl Cellulose Complete Media (R&D systems) containing SCF, IL-1, IL-6 and EPO, before spreading onto a 5 mL cell culture dish, which was subsequently incubated at 37°C with 5% CO_2_. Colonies were enumerated using an inverted microscope at 40 × magnification on days 4 and 8 after plating based on their morphology. The following colonies were analyzed: CFU-GEMM (colony forming unit − granulocyte, erythrocyte, macrophage, megakaryocyte), CFU-GM (colony forming unit − granulocyte, macrophage), CFU-G (colony forming unit − granulocyte), CFU-M (colony forming unit − macrophage), CFU-E (colony forming unit − erythroid), or BFU-E (burst forming unit − erythroid).

### Serum cytokine analysis

Mouse blood was allowed to clot at room temperature for 30 min, and serum obtained by centrifugation at 5000 × *g* for 10 min at 4°C and harvesting the supernatant. Cytokine and chemokine levels in the serum, including IFN-γ, TNF-α, IL-1β, IL-2, IL-4, IL-5, IL-6, IL-9, IL-10, IL-12, IL-13, IL-17A, IL-18, IL-22, IL-23, IL-27, CXCL1 (GRO-α), CXCL2 (MIP-2) and CXCL10 (IP-10), CCL2 (MCP-1), CCL3 (MIP-1α), CCL4 (MIP-1β), CCL5 (RANTES), CCL7 (MCP-3), CCL11 (Eotaxin) and GM-CSF were measured using a ProcartaPlex Mouse Cytokine and Chemokine Panel 1 kit (26 plex, eBioscience) per the manufacturer’s instructions. Serum EPO levels were quantified using a mouse EPO ELISA kit (Sigma-Aldrich) per manufacturer’s instructions.

### Statistical analysis

Statistical analysis was performed using Graph Pad Prism v8 (GraphPad Software, La Jolla California, USA). For mouse survival experiments, a Wilcoxon log-rank test was used, and for analysis of parasitemia and change in body weight a two-tailed student’s *t*-test was used. For other experiments, a one-way ANOVA with Šidák correction for multiple testing was used to compare between groups. A *p* value <0.05 was considered statistically significant.

## Results

### *Cish* ablation leads to alteration in blood cell parameters during infection

**Figure 1:**
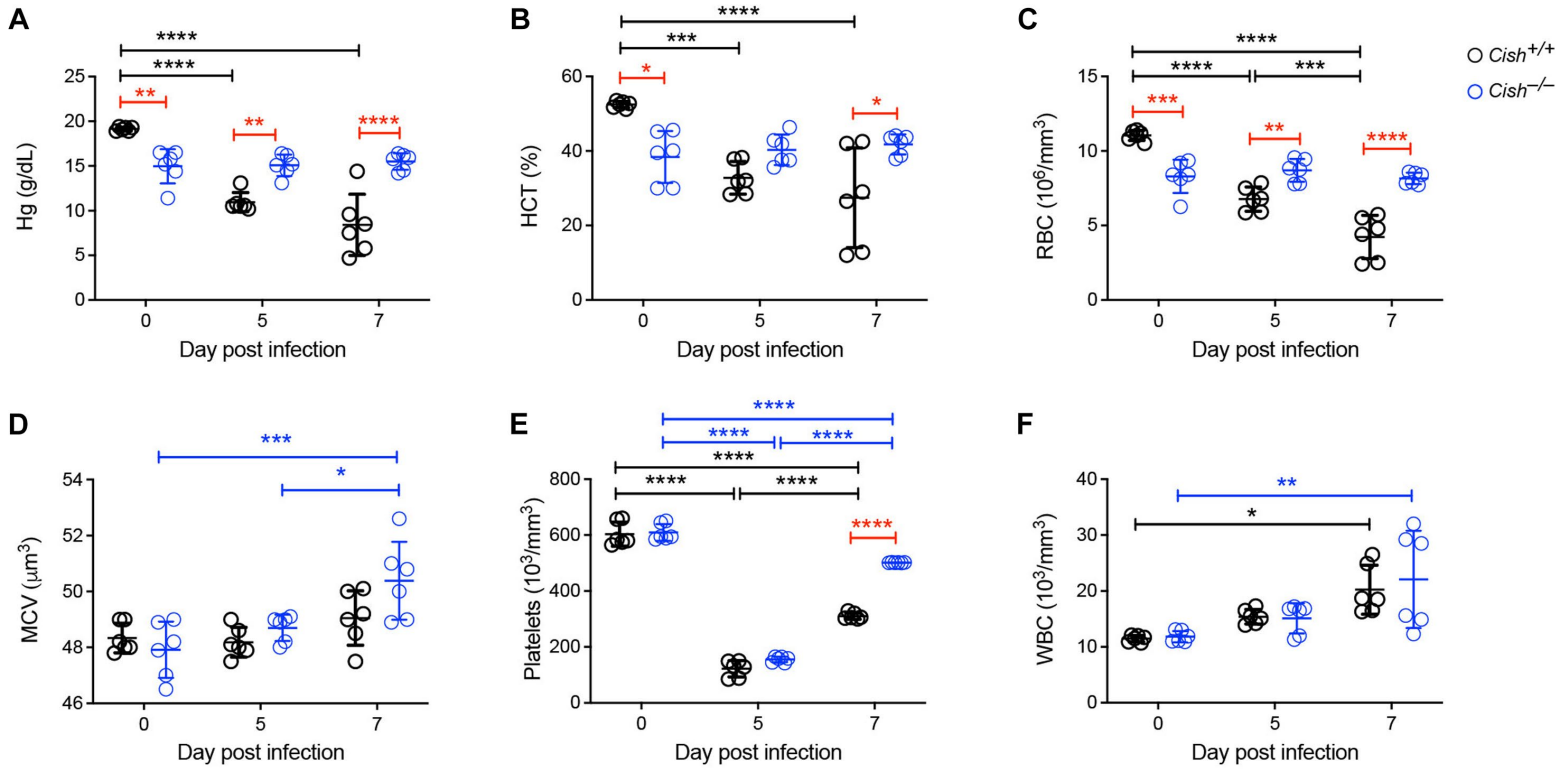
Impact of CISH ablation on blood parameters during a *Plasmodium berghei* infection. Peripheral blood harvested prior to infection of BALB/c *Cish*^+/+^ and *Cish*^−/−^ mice and at the indicated times post-infection were analyzed for **(A)** hemoglobin (Hg), **(B)** hematocrit (HCT), **(C)** red blood cell (RBC) count, **(D)** mean corpuscular volume (MCV), **(E)** platelet count, and **(F)** white blood cell (WBC) count. Data represents the mean ± SD (*n* = 6). Statistical significance between the indicated groups was determined by a one-way ANOVA. *p < 0.05, **p < 0.01, ***p < 0.001, ****p < 0.0001.

To assess the impact of *Cish* ablation on malaria infection, *Cish*^+/+^ and *Cish*^−/−^ mice on a BALB/c background were inoculated with 1 × 10^6^ *P. berghei* ANKA and at 0, 5 and 7 days post-infection (dpi) peripheral blood was collected. In agreement with previous work (Lakkavaram et al., 2020), the hemoglobin levels, hematocrit and RBC numbers of *Cish*^+/+^ mice significantly decreased as the infection progressed to less than half those of uninfected mice, consistent with the development of severe anemia (Figures 1A–C). In contrast, while hemoglobin levels, hematocrit and RBC numbers in uninfected *Cish*^−/−^ mice were significantly lower than in wildtype mice initially, they remained stable throughout the course of infection such that by 7 dpi they were significantly higher than *Cish*^+/+^ mice. No significant change was observed in the mean corpuscular volume of *Cish* ^+/+^ mice over the course of infection, although it was elevated at 7 dpi in *Cish*^−/−^ mice (Figure 1D). Platelet counts were comparable in uninfected mice of both genotypes and decreased to a similar extent by 5 dpi, but platelet count recovery was significantly stronger in *Cish*^−/−^ mice (Figure 1E). The number of WBCs in *Cish*^+/+^ mice increased during the course of infection as described (Lakkavaram et al., 2020), with similar results obtained with *Cish*^−/−^ mice (Figure 1F).

### Impact of *Cish* ablation on hematopoiesis during infection

**Figure 2:**
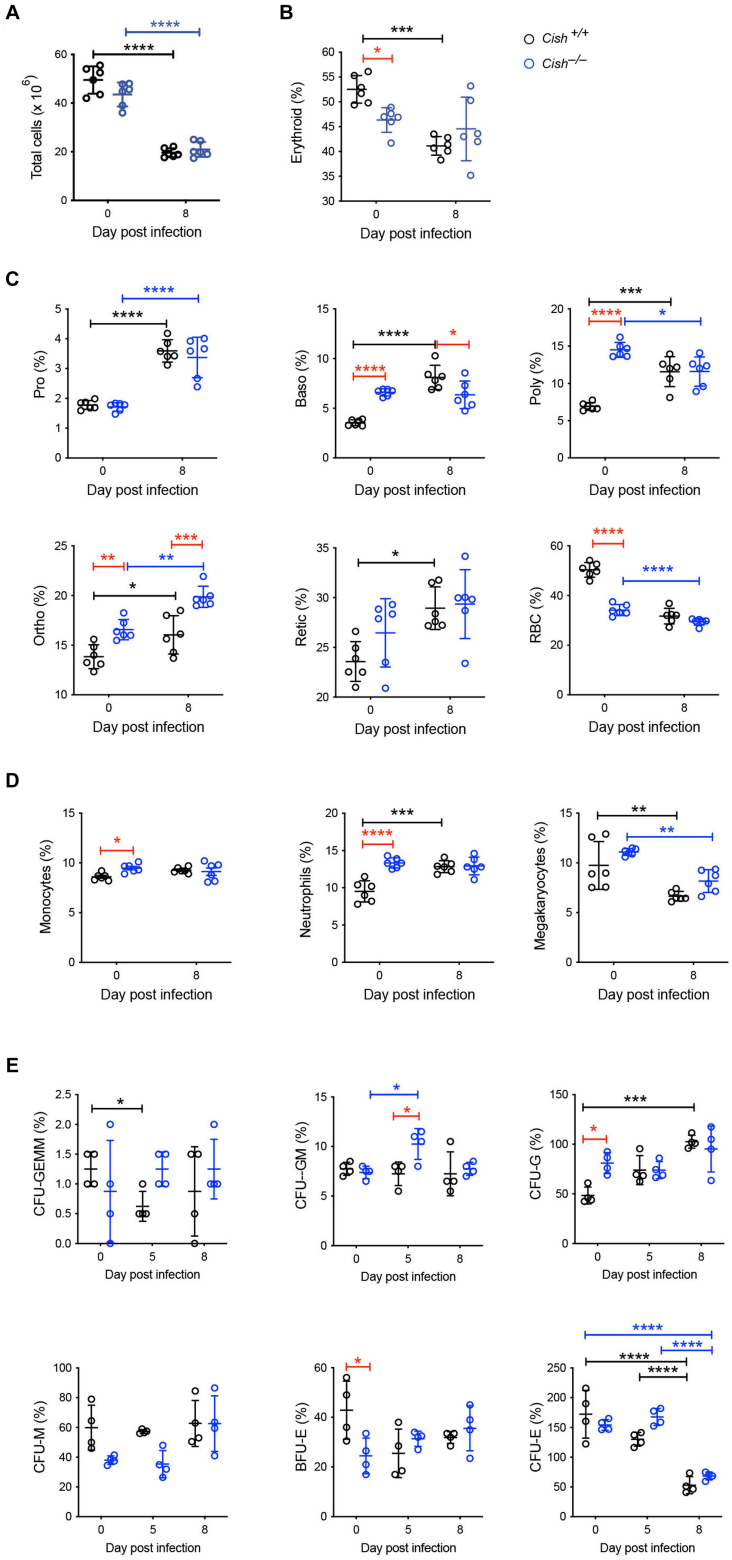
Impact of CISH ablation on hematopoiesis dynamics in the bone marrow of *P. berghei*-infected mice. Analysis of **(A)** bone marrow cellularity and relative proportions of **(B)** Ter119^+^ cells, **(C)** specific erythroid cell populations, **(D)** CD11b^+^Gr1^+^Ly6^−^ monocyte, CD11b^+^Gr1^+^Ly6^+^ neutrophil, and CD61^+^ megakaryocyte cells, and **(E)** hematopoietic colony forming cells during a *P. berghei* infection of BALB/c *Cish*^+/+^ and *Cish*^−/−^ mice. Data represent individual mice and the mean ± SD (*n* = 6 mice). Statistical significance between the indicated groups was determined by a one-way ANOVA. *p < 0.05, **p < 0.01, ***p < 0.001, ****p < 0.0001. Pro, proerythroblasts; Baso, basophilic erythroblasts; Poly, polychromatic erythroblasts; Ortho, orthochromatic erythroblasts; Retic, reticulocytes; RBC, red blood cells; CFU, colony forming units; CFU-GEMM, CFU, granulocyte, erythrocyte, megakaryocyte; CFU-GM, CFU, granulocyte, macrophage; CFU-G, CFU, granulocyte; CFU-M, CFU, macrophage; CFU-E, CFU, erythroid; BFU-E, burst forming unit – erythroid.

**Figure 3:**
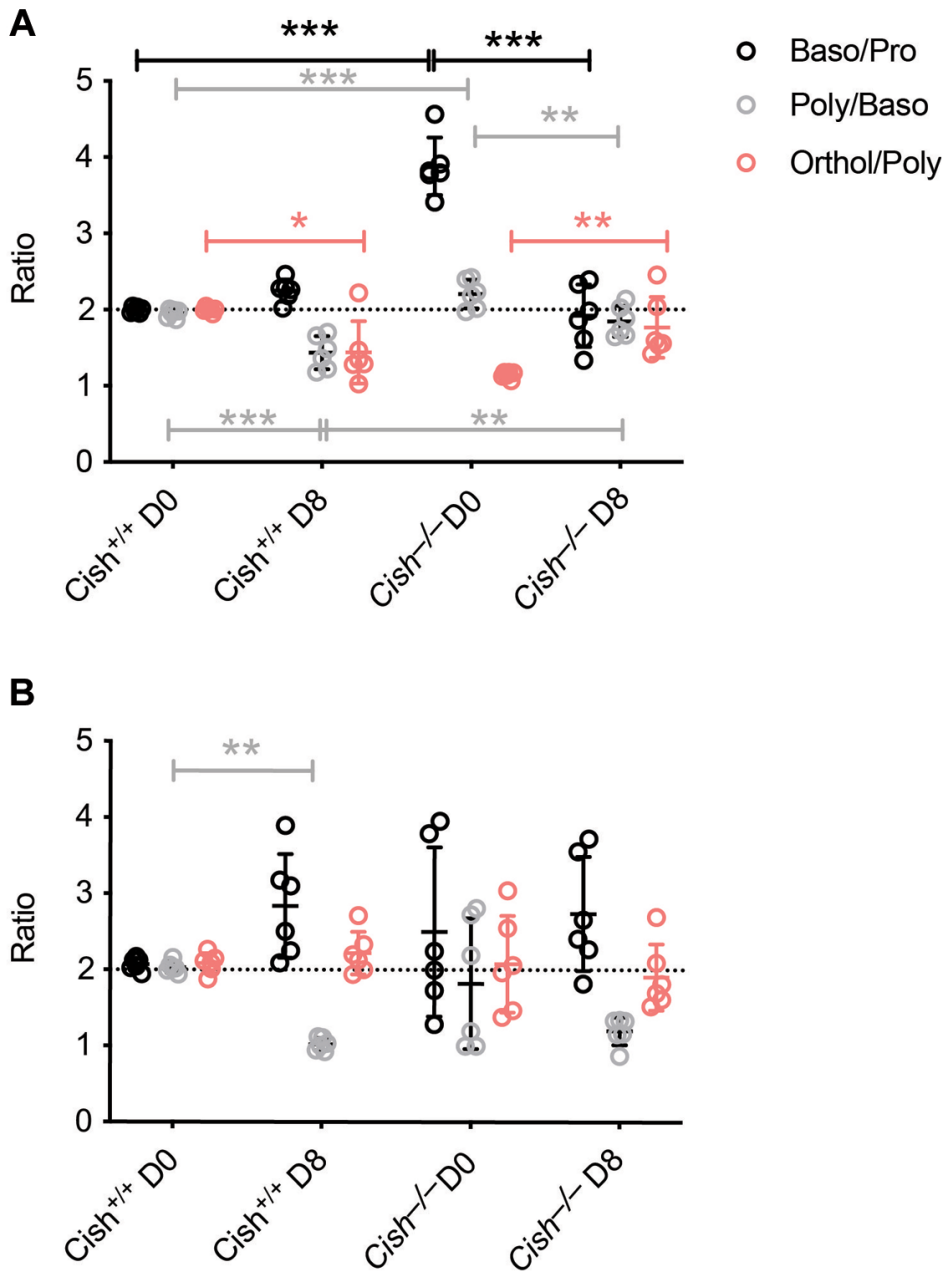
Comparison of terminal erythroid differentiation before and after *P. berghei* infection. The ratio of the indicated erythroblast populations in the **(A)** bone marrow and **(B)** spleen in response to infection of BALB/c *Cish*^+/+^ and *Cish*^−/−^ mice. Normal differentiation involving a doubling of erythroblast number with each successive mitosis is represented by the dotted line.

The impact of *Cish* ablation on bone marrow and spleen hematopoiesis during a *P. berghei* infection was assessed. No difference in bone marrow cellularity was observed between BALB/c *Cish*^+/+^ and *Cish*^−/−^ mice before or after infection, with the cellularity decreasing to a similar extent in each case (Figure 2A; Supplementary Figure S1). Infection resulted in a significant decline in the frequency and number of Ter119^+^ erythroid cells in *Cish*^+/+^ mice (Figure 2B; Supplementary Figure S2). This was not the case for *Cish*^−/−^ mice, so although they had a significantly lower frequency and number of Ter119^+^ erythroid cells compared to *Cish*^+/+^ mice prior to infection and their numbers declined during infection, at 8 dpi the difference in cell frequency and number between genotypes was no longer significant (Figure 2B; Supplementary Figure S2). Ter119/CD44 double staining (Chen et al., 2009) was used to quantify the frequency and number of specific stages of erythropoietic development (Supplementary Figures S1, S2; Figure 2C). Following infection, a statistically significant increase in the frequency of all erythroblast populations and reticulocytes was seen in *Cish* ^+/+^ mice, while the RBC frequency decreased, although this did not reach statistical significance (Figure 2C). As a consequence, the ratio of pro-:basophilic:poly-chromatic:orthochromatic erythroblasts did not follow the usual doubling of cell number that occurs as a result of each successive mitosis in infected *Cish^+/+^* mice (Figure 3A). Uninfected BALB/c *Cish*^−/−^ mice possessed a significantly increased frequency of basophilic, polychromatic and orthochromatic erythroblasts compared to *Cish*^+/+^ mice, while RBC frequency was decreased. In response to infection, only the frequency of proerythroblasts and orthochromatic erythroblasts increased significantly in *Cish*^−/−^ mice, while in contrast the polychromatic erythroblasts and RBC significantly decreased. As such, the frequency of basophilic erythroblasts became significantly lower and orthochromatic erythroblasts significantly elevated compared to infected *Cish* ^+/+^ mice (Figure 2C). Thus, the ratios and numbers of erythroblast populations, while perturbed in uninfected BALB/c *Cish*^−/−^ mice, were close to normal after infection and similar in number to *Cish^+/+^* mice (Figure 3A; Supplementary Figure S2).

Infection resulted in a significant increase in CD11b^+^Gr1^+^Ly6G^+^ neutrophils in *Cish*^+/+^ mice but not CD11b^+^Gr1^+^Ly6G^−^ monocytes. While the frequency of the CD11b^+^Gr1^+^Ly6G^−^ monocyte lineage and the frequency and total number of CD11b^+^Gr1^+^Ly6G^+^ neutrophil lineages were significantly increased in uninfected *Cish*^−/−^ mice compared to equivalent *Cish*^+/+^ mice (Figure 2D; Supplementary Figures S2, S3), these populations did not change drastically in infected *Cish*^−/−^ mice, such that at 8 dpi they were comparable in frequency and number between mouse genotypes. The frequency and number of CD61^+^ cells significantly declined in both genotypes to a similar extent (Figure 2D; Supplementary Figures S2, S3).

Given the differences observed in hematopoiesis between BALB/c *Cish*^+/+^ and *Cish*^−/−^ mice, the frequency of hematopoietic progenitor cells was also analyzed. Infection of *Cish*^+/+^ mice resulted in a transient significant decrease in CFU-GEMM, a sustained increase in CFU-G and a decrease in CFU-E (Figure 2E). Uninfected *Cish*^−/−^ mice possessed a significantly higher frequency of CFU-G but a lower frequency of BFU-E compared to *Cish*^+/+^ mice. However, the only precursors that significantly increased in frequency in the infected *Cish*^−/−^ mice were CFU-GM at 5 dpi when they were also higher than in *Cish*^+/+^ mice, but there was a decline in CFU-E frequency at 8 dpi (Figure 2E).

**Figure 4:**
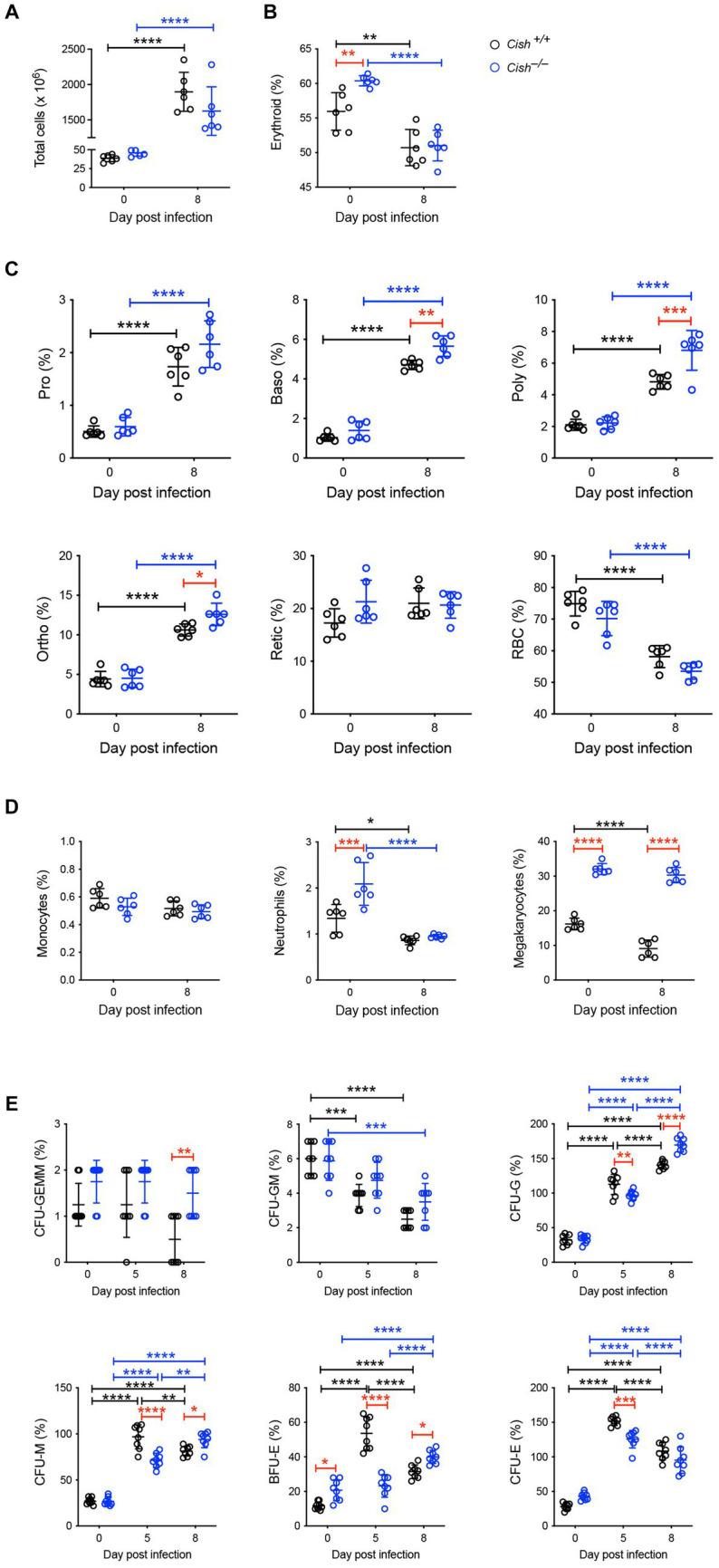
Impact of CISH ablation on hematopoiesis dynamics in the spleen of *P. berghei*-infected mice. Analysis of **(A)** spleen cellularity, and relative proportion of **(B)** Ter119^+^ cells, **(C)** specific erythroid populations, **(D)** CD11b^+^Gr1^+^Ly6^−^ monocyte, CD11b^+^Gr1^+^Ly6^+^ neutrophil, and CD61^+^ megakaryocytic cells, and **(E)** hematopoietic colony forming cells during *P. berghei* infection of BALB/c *Cish*^+/+^ and *Cish*^−/−^ mice. Data represent individual mice and the mean ± SD (*n* = 6 mice). Statistical significance between the indicated groups was determined by a one-way ANOVA. *p < 0.05, **p < 0.01, ***p < 0.001, ****p < 0.0001.

In the spleen a similar increase in cellularity after infection of both *Cish*^+/+^ and *Cish*^−/−^ mice was observed, with the increase in total numbers of Ter119^+^ erythroid cells also comparable (Figure 4A; Supplementary Figures S1, S2). However, there was a significant decline in the frequency of Ter119^+^ erythroid cells in both genotypes; the decrease in *Cish*^−/−^ mice was more pronounced from its initially elevated basal level, such that frequency of Ter119^+^ erythroid cells at 8 dpi was now comparable between genotypes (Figure 4B; Supplementary Figure S1). Infection led to a significant increase in the frequency of all erythroblast populations and a decrease in RBC in both genotypes, but basophilic, polychromatic and orthochromatic erythroblasts all increased to a greater extent in *Cish*^−/−^ mice such that they were significantly higher than in *Cish*^+/+^ mice at 8 dpi (Figure 4C; Supplementary Figure S1). The number of polychromatic erythroblasts was also significantly higher in the *Cish*^−/−^ mice at this time (Supplementary Figure S2). However, the ratios of erythroblast populations were relatively normal, with only the polychromatic:basophilic erythroblast ratio significantly altered in *Cish*^+/+^ mice (Figure 3B).

Infection resulted in no change in the frequency of splenic CD11b^+^Gr1^+^Ly6G^−^ monocytes in either mouse genotype. The frequency of CD11b^+^Gr1^+^Ly6G^+^ neutrophils was basally elevated in *Cish*^−/−^ mice, but significantly decreased in both genotypes with infection, such that at 8 dpi their frequency was comparable (Figure 4D; Supplementary Figure S3). The frequency of CD61^+^ cells was significantly decreased in *Cish*^+/+^ mice only and was significantly lower than in *Cish*^−/−^ mice pre- and post-infection, and the number of CD61^+^ cells in *Cish*^+/+^ mice at 8 dpi was also significantly lower (Figure 4D; Supplementary Figure S2).

Infection led to a significant decrease in the frequency of splenic CFU-GEMM in *Cish*^+/+^ mice only and a significant decrease in the frequency of CFU-GM in both genotypes, although more rapidly in *Cish*^+/+^ mice (Figure 4E). Conversely, a significant increase was observed in the frequency of CFU-G, CFU-M, BFU-E and CFU-E in both genotypes. This occurred more rapidly in *Cish*^+/+^ mice such that their frequencies were significantly higher than in the *Cish*^−/−^ mice at 5 dpi, but by 8 dpi the frequency of all these populations except for CFU-E was significantly higher in the *Cish*^−/−^ mice, with this being the case basally just for BFU-E (Figure 4E).

As EPO is a key mediator of erythropoiesis (Jelkmann, 2007), serum EPO levels were also analyzed in *Cish*^+/+^ and *Cish*^−/−^ mice. Infection resulted in significantly elevated EPO expression at 8 dpi in *Cish*^+/+^ mice, but not in the *Cish*^−/−^ mice from a similar basal level (Table 1).

**Table 1:** Impact of *P. berghei* infection on serum EPO levels.

	*Cish^+/+^* (mean ± SD)	*Cish^−/−^* (mean ± SD)	*p* value
Day 0	8.83 ± 4.16 pg./mL	6.09 ± 6.46 pg./mL	*p* > 0.9999
Day 8	118.60 ± 66.53 pg./mL	25.77 ± 17.18 pg./mL	*p* = 0.0001

### *Cish* ablation does not influence the outcome of *Plasmodium berghei* infection

**Figure 5:**
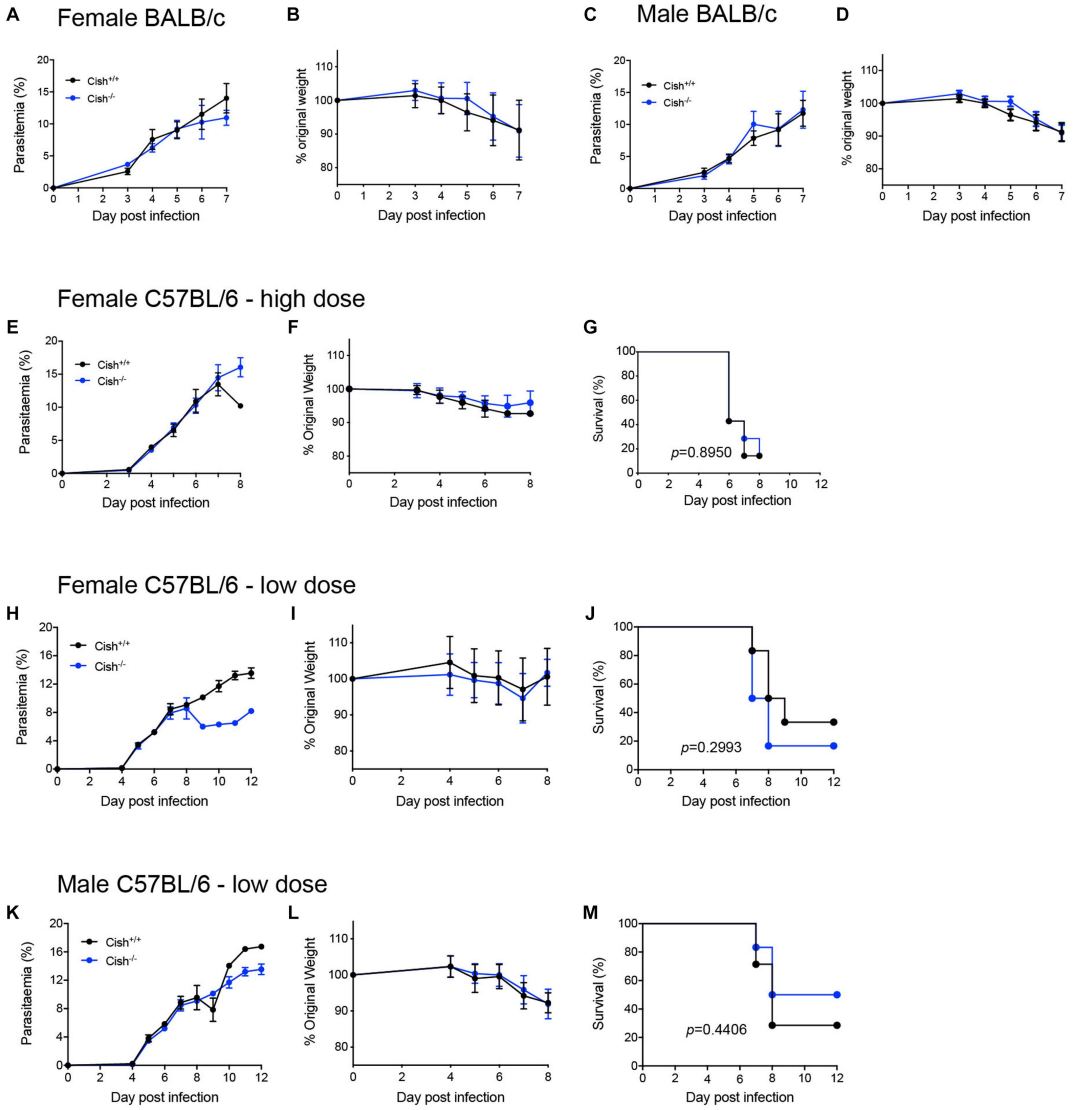
Deletion of CISH does not affect the outcome of *P. berghei* infection. Analysis of **(A)** course of parasitemia and **(B)** weight loss in female BALB/c *Cish*^+/+^ and *Cish*^−/−^ mice (*n* = 10 mice) and **(C)** course of parasitemia and **(D)** weight loss in male BALB/c *Cish*^+/+^ and *Cish*^−/−^ mice (*n* = 10) after infection with 1 × 10^6^ *P. berghei-*infected RBC. Analysis of **(E)** course of parasitemia, **(F)** weight loss, and **(G)** survival of female C57BL/6 *Cish*^+/+^ (*n* = 7) and *Cish*^−/−^mice (*n* = 7) after infection with 1 × 10^6^ *P. berghei*-infected RBC. Analysis of **(H)** course of parasitemia, **(I)** weight loss, and **(J)** survival of female C57BL/6 *Cish*^+/+^ (*n* = 6) and *Cish* ^−/−^mice (*n* = 5) or **(K)** course of parasitemia, **(L)** weight loss, and **(M)** survival of male C57BL/6 *Cish*^+/+^ (*n* = 7) and *Cish* ^−/−^mice (*n* = 6) after infection with 1 × 10^5^ *P. berghei*-infected RBC. Statistical analysis of parasitemia and weight loss were performed using a two-sided unpaired *t*-test, and of survival using a log-rank test.

To determine whether the differences in hematopoiesis impacted malaria infection, both female and male BALB/c *Cish*^+/+^ and *Cish*^−/−^ littermates were infected by intraperitoneal injection with 1 × 10^6^ *P. berghei* ANKA-infected RBCs. No significant differences in parasitemia levels or kinetics (Figures 5A,C) were observed between the genotypes nor in the amount of weight lost as a result of infection of either sex (Figures 5B,D).

**Figure 6:**
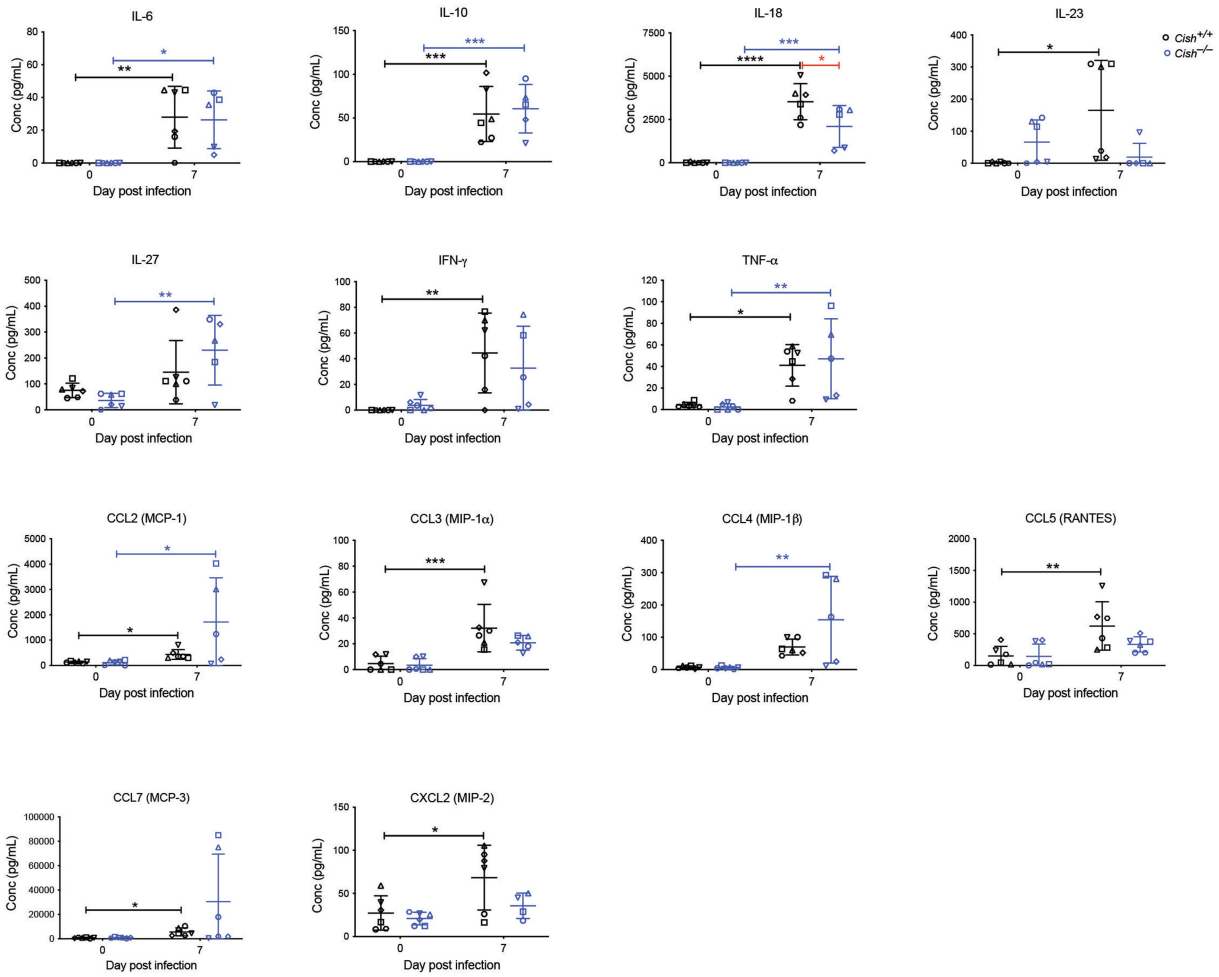
Analysis of cytokines and chemokines in *P. berghei* infection. Concentrations of the indicated cytokines and chemokines that were either significantly different between genotypes or were significantly altered in response to a *P. berghei* infection in BALB/c *Cish*^+/+^ and *Cish* ^−/−^ mice. Data represent individual mice and the mean ± SD (*n* = 6 mice). Statistical significance between the indicated groups was determined by an unpaired *t*-test. *p < 0.05, **p < 0.01, ***p < 0.001, ****p < 0.0001.

Cytokines and chemokines play a significant role during a *Plasmodium* infection (Angulo and Fresno, 2002; Ioannidis et al., 2014). Therefore, a large set of these were analyzed before and after infection. Female *Cish*^+/+^ and *Cish*^−/−^ mice displayed similar basal levels of all cytokines and chemokines (Figure 5), but infection resulted in a significant increase in IL-6, IL-10, IL-18, IL-23, IFN-γ, TNF-α, CCL2, CCL3, CCL5, CCL7, CXCL2, and CXCL10 in *Cish*^+/+^ mice, whereas *Cish*^−/−^ mice exhibited a slightly different response; the only significant increases observed in the *Cish*^−/−^ mice were for IL-6, IL-10, IL-18, TNF-α, CCL2, although IL-27 and CCL4 levels were additionally significantly elevated in contrast to *Cish*^+/+^ mice. Despite this, the only cytokine for which there was a significant difference between the mice genotypes after infection was IL-18, being higher in *Cish*^+/+^ mice (Figure 6).

The potential contribution of CISH to ECM was also investigated using the alternative C57BL/6 model, with female *Cish*^+/+^ and *Cish*^−/−^ littermates on this background infected with 1 × 10^6^ *P. berghei* ANKA-infected RBCs and examining parasitemia, weight loss and ECM susceptibility. No significant differences were observed between genotypes in any of these parameters, with mice from both genotypes succumbing to ECM from as early as 6 dpi (Figures 5E–G). A lower dose of 1 × 10^5^ *P. berghei* infected RBCs was also administered to delay the patency and onset of ECM, this time to both female (Figures 5H–J) and male (Figures 5K–M) mice. However, no significant difference between the genotypes was observed for parasitemia and loss of bodyweight for either sex to 8 dpi (Figures 5H,I,K,L), noting that beyond this time the low number of mice remaining prevented statistical comparison. However, there was no significant difference in the survival rates between mouse genotypes for either sex (Figures 5J,M).

## Discussion

This study examined how the absence of CISH affected hematopoietic, cytokine and blood cell parameters of mice in response to acute infection with *P. berghei*. Uninfected BALB/c *Cish^−/−^* mice exhibited a notable difference in basal bone marrow erythropoiesis, with a lower frequency of BFU-E compared to *Cish^+/+^* mice, as well as an altered frequency, number and ratio of specific erythroblast populations, likely underpinning the reduction in RBCs and diminished frequency of Ter119^+^ erythroid cells in the bone marrow. In contrast, erythropoiesis in the spleens of uninfected *Cish ^−/−^* mice showed normal ratios, with the expected physiological progression of erythroid differentiation. However, as the bone marrow is mostly responsible for basal erythropoiesis, the *Cish^−/−^* mice exhibited reduced peripheral RBC count, hemoglobin levels and hematocrit. These results are in agreement with recent research on the role of CISH in normal erythropoiesis, with suppression of bone marrow erythropoiesis associated with altered expression of a set of erythroid-related genes observed in *Cish*^−/−^ mice, but increased proliferation in the spleen (Maymand et al., 2023). Uninfected *Cish*^−/−^ mice also showed a significantly increased frequency of CFU-G in the bone marrow, consistent with elevated frequency of CD11b^+^Gr1^+^Ly6G^+^ neutrophil cells in this organ, while in the spleen, the frequency of CD11b^+^Gr1^+^Ly6G^+^ neutrophils was similarly increased. These findings are consistent with a recent study examining the role of CISH in myelopoiesis (Naser et al., 2023). In addition, CD61^+^ cells of the megakaryocytic lineage were also elevated in the spleen of *Cish ^−/−^* mice.

Intriguingly, despite dysregulated basal erythropoiesis in uninfected *Cish^−/−^* mice, they were able to maintain relatively stable peripheral blood counts and hematocrit during infection. This correlated with the ability to maintain BFU-E and a normal pattern of erythroid differentiation in the bone marrow, although the frequency of CFU-E did decline in a similar manner to infected *Cish^+/+^* mice. In contrast, the orthochromatic:polychromatic erythroblast ratio could not be maintained in the *Cish*^+/+^ mice. Spleen erythropoiesis was less impacted in infected *Cish*^−/−^ mice, but *Cish*^+/+^ mice exhibited a statistically significant decrease in the polychromatic:basophilic ratio compared to uninfected mice. The differences in basal erythropoiesis between the genotypes appeared to be independent of EPO, since uninfected *Cish^+/+^* and *Cish^−/−^* mice exhibited similar levels of EPO. An increase in EPO levels was observed in infected *Cish^+/+^* but not *Cish^−/−^* mice, most likely because severe anemia was observed only in *Cish^+/+^* mice. We have recently shown that CISH expression is induced by EPO in both bone marrow and spleen. *Cish^−/−^* mice could also respond robustly overall to EPO injection, with bone marrow erythropoiesis somewhat blunted but with spleen erythropoiesis enhanced to compensate (Maymand et al., 2023). Collectively this indicates a differential impact of CISH ablation on erythropoiesis at these two sites and suggests that CISH acts as an inducible regulator of EPO signaling *in vivo.*

While there were clear differences in hematopoietic parameters between *Cish* genotypes, similar parasitemia kinetics were observed in infected female BALB/c *Cish*^+/+^ and *Cish*^−/−^ mice, which also lost a comparable amount of bodyweight. Male BALB/c *Cish*^+/+^ and *Cish*^−/−^ mice likewise exhibited equivalent infection outcomes, although hematopoietic parameters were not examined in these mice. Both female *Cish* genotypes exhibited similar elevations of specific cytokines, including those associated with anemia, such as TNF-α and IL-6 (Lyke et al., 2004; Chopra et al., 2015), with only IL-18 levels higher in *Cish*^+/+^ mice following infection. These results are consistent with there being a similar frequency of CD11b^+^Gr1^+^Ly6G^−^ monocyte and CD11b^+^Gr1^+^Ly6G^+^ neutrophil lineages in both genotypes at 8dpi.

In the C57BL/6 model of ECM, female *Cish*^+/+^ and *Cish*^−/−^ mice displayed a similar course of parasitemia, change in bodyweight and kinetics of ECM induction after being infected with *P. berghei,* which was also seen in male *Cish*^+/+^ and *Cish*^−/−^ mice. The inflammatory responses in *P. berghei* infected mice that succumb to ECM has been well studied, with CD4^+^ T cells (Yanez et al., 1996), CD8^+^ T cells (Nitcheu et al., 2003) and NK cells (Hansen et al., 2007), all previously shown to play a role in the development of ECM. Studies have shown the absence of CISH leads to an enhancement of NK cell survival and proliferation (Delconte et al., 2016) and CD8^+^ T cell expansion and cytokine polyfunctionality (Palmer et al., 2015; Delconte et al., 2016). However, both *Cish*^+/+^ and *Cish*^−/−^ mice suffered a similar fate following infection with *P. berghei* despite this. It is important to note that in the absence of CISH, other SOCS proteins that also contribute to the negative regulation of cytokine signaling (Sobah et al., 2021), could potentially compensate to control the inflammatory responses that drive ECM. For example, SOCS2 and SOCS3 expression was found to elevated in the arcuate nucleus of *Cish*^−/−^ mice, which suggests that compensatory mechanisms exist (Naser et al., 2022). Therefore, it would be interesting to examine whether the expression of other SOCS proteins in the bone marrow and in regions of the brain where parasites sequester is affected in the absence of CISH.

Collectively these results indicate that CISH in not a crucial contributor of parasite load and that an absence of CISH alone is unable to influence the outcome of an acute malaria infection. These findings, therefore, fail to explain the association between SNPs in human *CISH* and increased susceptibility to the severe manifestations of malaria infections (Khor et al., 2010). However, complete ablation of CISH is not the same as the more subtle impacts seen in the human gene that instead results in altered expression, and so efforts to recapitulate the relevant human SNPs in mice would be worthwhile. It is also likely that prior exposure of human participants to *Plasmodium* and other infectious agents may influence how CISH impacts the immune response compared to naïve mice. Indeed, the loss of CISH has been shown to lead to dysregulated immune responses to different pathogens (Sun et al., 2014; Kotas et al., 2021). It would therefore be interesting to examine whether repeated exposure of *Cish*^−/−^ mice to *Plasmodium* dysregulates the immune response in a manner that might enhance the severity of disease. In addition, chronic infection likely places more stress on the maintenance of erythropoiesis and so investigation of whether the dysregulated basal erythropoietic response in *Cish*^−/−^ mice affects the progression of such an infection would also be worthwhile.

## Data availability statement

The original contributions presented in the study are included in the article/Supplementary material, further inquiries can be directed to the corresponding author.

## Author contributions

AL: Formal analysis, Investigation, Writing – original draft, Methodology. SM: Investigation, Writing – review & editing. WN: Investigation, Writing – review & editing. AW: Conceptualization, Validation, Writing – review & editing. TdK-W: Conceptualization, Data curation, Formal analysis, Funding acquisition, Investigation, Resources, Supervision, Writing – original draft.

## References

[ref1] Adams T. E. Hansen J. A. Starr R. Nicola N. A. Hilton D. J. Billestrup N. (1998). Growth hormone preferentially induces the rapid, transient expression of Socs-3, a novel inhibitor of cytokine receptor signaling. J. Biol. Chem. 273, 1285–1287. doi: 10.1074/jbc.273.3.1285 9430658

[ref2] Aman M. J. Migone T. S. Sasaki A. Ascherman D. P. Zhu M. Soldaini E.. (1999). CIS associates with the interleukin-2 receptor beta chain and inhibits interleukin-2-dependent signaling. J. Biol. Chem. 274, 30266–30272. doi: 10.1074/jbc.274.42.30266, PMID: 10514520

[ref3] Angulo I. Fresno M. (2002). Cytokines in the pathogenesis of and protection against malaria. Clin. Diagn. Lab. Immunol. 9, 1145–1152. doi: 10.1128/cdli.9.6.1145-1152.2002 12414742 PMC130117

[ref4] Beare N. A. V. Harding S. P. Taylor T. E. Lewallen S. Molyneux M. E. (2009). Perfusion abnormalities in children with cerebral malaria and malarial retinopathy. J. Infect. Dis. 199, 263–271. doi: 10.1086/595735 18999956 PMC2757304

[ref5] Chen K. Liu J. Heck S. Chasis J. A. An X. Mohandas N. (2009). Resolving the distinct stages in erythroid differentiation based on dynamic changes in membrane protein expression during erythropoiesis. Proc. Natl. Acad. Sci. U. S. A. 106, 17413–17418. doi: 10.1073/pnas.0909296106 19805084 PMC2762680

[ref6] Chopra M. Langenhorst D. Beilhack A. Serfling E. Patra A. K. (2015). Interleukin-2 critically regulates bone marrow erythropoiesis and prevents anemia development. Eur. J. Immunol. 45, 3362–3374. doi: 10.1002/eji.201545596 26404745

[ref7] Craig A. G. Grau G. E. Janse C. Kazura J. W. Milner D. Barnwell J. W.. (2012). The role of animal models for research on severe malaria. PLoS Pathog. 8:e1002401. doi: 10.1371/journal.ppat.1002401 22319438 PMC3271056

[ref8] De Oca M. M. Engwerda C. Haque A. (2013). *Plasmodium berghei* Anka (PbA) infection of C57bl/6J mice: a model of severe malaria. Methods Mol. Biol. 1031, 203–213. doi: 10.1007/978-1-62703-481-4_23 23824903

[ref9] Delconte R. B. Kolesnik T. B. Dagley L. F. Rautela J. Shi W. Putz E. M.. (2016). CIS is a potent checkpoint in NK cell-mediated tumor immunity. Nat. Immunol. 17, 816–824. doi: 10.1038/ni.3470 27213690

[ref10] Dorovini-Zis K. Schmidt K. Huynh H. Fu W. Whitten R. O. Milner D.. (2011). The neuropathology of fatal cerebral malaria in malawian children. Am. J. Pathol. 178, 2146–2158. doi: 10.1016/j.ajpath.2011.01.016 21514429 PMC3081150

[ref11] Driss A. Hibbert J. M. Wilson N. O. Iqbal S. A. Adamkiewicz T. V. Stiles J. K. (2011). Genetic polymorphisms linked to susceptibility to malaria. Malar. J. 10:271. doi: 10.1186/1475-2875-10-271 21929748 PMC3184115

[ref12] Francischetti I. M. Seydel K. B. Monteiro R. Q. (2008). Blood coagulation, inflammation, and malaria. Microcirculation 15, 81–107. doi: 10.1080/10739680701451516 18260002 PMC2892216

[ref13] Hansen D. S. Bernard N. J. Nie C. Q. Schofield L. (2007). NK cells stimulate recruitment of CXCR3+ T cells to the brain during *Plasmodium berghei*-mediated cerebral malaria. J. Immunol. 178, 5779–5788. doi: 10.4049/jimmunol.178.9.5779, PMID: 17442962

[ref14] Hanum P. S. Hayano M. Kojima S. (2003). Cytokine and chemokine responses in a cerebral malaria-susceptible or -resistant strain of mice to *Plasmodium berghei* Anka infection: early chemokine expression in the brain. Int. Immunol. 15, 633–640. doi: 10.1093/intimm/dxg065 12697663

[ref15] Helman D. Sandowski Y. Cohen Y. Matsumoto A. Yoshimura A. Merchav S.. (1998). Cytokine-inducible Sh2 protein (CIS3) and JAK2 binding protein (JAB) abolish prolactin receptor-mediated Stat5 signaling. FEBS Lett. 441, 287–291. doi: 10.1016/S0014-5793(98)01555-5 9883901

[ref16] Hunter M. G. Jacob A. O’Donnell L. C. Agler A. Druhan L. J. Coggeshall K. M.. (2004). Loss of ship and CIS recruitment to the granulocyte colony-stimulating factor receptor contribute to hyperproliferative responses in severe congenital neutropenia/acute myelogenous leukemia. J. Immunol. 173, 5036–5045. doi: 10.4049/jimmunol.173.8.5036, PMID: 15470047

[ref17] Ioannidis L. J. Nie C. Q. Hansen D. S. (2014). The role of chemokines in severe malaria: more than meets the eye. Parasitology 141, 602–613. doi: 10.1017/S0031182013001984, PMID: 24476686 PMC3962270

[ref18] Jelkmann W. (2007). Erythropoietin after a century of research: younger than ever. Eur. J. Haematol. 78, 183–205. doi: 10.1111/j.1600-0609.2007.00818.x, PMID: 17253966

[ref19] Ji L. D. Xu W. N. Chai P. F. Zheng W. Qian H. X. Xu J. (2014). Polymorphisms in the CISH gene are associated with susceptibility to tuberculosis in the Chinese Han population. Infect. Genet. Evol. 28, 240–244. doi: 10.1016/j.meegid.2014.10.006 25460819

[ref20] Khor C. C. Vannberg F. O. Chapman S. J. Guo H. Wong S. H. Walley A. J.. (2010). CISH and susceptibility to infectious diseases. N. Engl. J. Med. 362, 2092–2101. doi: 10.1056/NEJMoa0905606, PMID: 20484391 PMC3646238

[ref21] Kotas M. E. Mroz N. M. Koga S. Liang H. E. Schroeder A. W. Ricardo-Gonzalez R. R.. (2021). CISH constrains the tuft-ILC2 circuit to set epithelial and immune tone. Mucosal Immunol. 14, 1295–1305. doi: 10.1038/s41385-021-00430-6 34290377 PMC8528700

[ref22] Lakkavaram A. Lundie R. J. Do H. Ward A. C. De Koning-Ward T. F. (2020). Acute *Plasmodium berghei* mouse infection elicits perturbed erythropoiesis with features that overlap with anemia of chronic disease. Front. Microbiol. 11:702. doi: 10.3389/fmicb.2020.00702 32373101 PMC7176981

[ref23] Lamikanra A. A. Brown D. Potocnik A. Casals-Pascual C. Langhorne J. Roberts D. J. (2007). Malarial anemia: of mice and men. Blood 110, 18–28. doi: 10.1182/blood-2006-09-018069 17341664

[ref24] Li S. Chen S. Xu X. Sundstedt A. Paulsson K. M. Anderson P.. (2000). Cytokine-induced SRC homology 2 protein (CIS) promotes T cell receptor-mediated proliferation and prolongs survival of activated T cells. J. Exp. Med. 191, 985–994. doi: 10.1084/jem.191.6.985 10727460 PMC2193118

[ref25] Liu X. H. Xu S. B. Yuan J. Li B. H. Zhang Y. Yuan Q.. (2009). Defective interleukin-4/Stat6 activity correlates with increased constitutive expression of negative regulators SOCS-3, SOCS-7, and CISH in colon cancer cells. J. Interf. Cytokine Res. 29, 809–816. doi: 10.1089/jir.2009.0004 19929568

[ref26] Louis C. Souza-Fonseca-Guimaraes F. Yang Y. D'silva D. Kratina T. Dagley L.. (2020). NK cell-derived GM-CSF potentiates inflammatory arthritis and is negatively regulated by CIS. J. Exp. Med. 217:e20191421. doi: 10.1084/jem.20191421 32097462 PMC7201918

[ref27] Lyke K. E. Burges R. Cissoko Y. Sangare L. Dao M. Diarra I.. (2004). Serum levels of the proinflammatory cytokines interleukin-1 beta (IL-1beta), IL-6, IL-8, IL-10, tumor necrosis factor alpha, and IL-12(p70) in Malian children with severe *Plasmodium falciparum* malaria and matched uncomplicated malaria or healthy controls. Infect. Immun. 72, 5630–5637. doi: 10.1128/IAI.72.10.5630-5637.2004, PMID: 15385460 PMC517593

[ref28] Macpherson G. G. Warrell M. J. White N. J. Looareesuwan S. Warrell D. A. (1985). Human cerebral malaria. A quantitative ultrastructural analysis of parasitized erythrocyte sequestration. Am. J. Pathol. 119, 385–401. 3893148 PMC1888001

[ref29] Marquet S. (2018). Overview of human genetic susceptibility to malaria: from parasitemia control to severe disease. Infect. Genet. Evol. 66, 399–409. doi: 10.1016/j.meegid.2017.06.001 28579526

[ref30] Matsumoto A. Masuhara M. Mitsui K. Yokouchi M. Ohtsubo M. Misawa H.. (1997). CIS, a cytokine inducible SH2 protein, is a target of the JAK-Stat5 pathway and modulates Stat5 activation. Blood 89, 3148–3154. doi: 10.1182/blood.V89.9.3148 9129017

[ref31] Maymand S. Lakkavaram A. L. Naser W. Rasighaemi P. Dlugolenski D. Liongue C.. (2023). Role of cytokine-inducible Sh2 domain-containing (CISH) protein in the regulation of erythropoiesis. Biomolecules 13:1510. doi: 10.3390/biom13101510 37892192 PMC10604548

[ref32] Moxon C. A. Gibbins M. P. Mcguinness D. Milner D. A. Jr. Marti M. (2020). New insights into malaria pathogenesis. Annu. Rev. Pathol. 15, 315–343. doi: 10.1146/annurev-pathmechdis-012419-032640 31648610

[ref33] Moxon C. A. Heyderman R. S. Wassmer S. C. (2009). Dysregulation of coagulation in cerebral malaria. Mol. Biochem. Parasitol. 166, 99–108. doi: 10.1016/j.molbiopara.2009.03.006, PMID: 19450727 PMC2724037

[ref34] Naderi M. Hashemi M. Safdari A. Bahari G. Taheri M. (2016). Association of genetic polymorphisms of CISH with the risk of pulmonary tuberculosis in Zahedan, Southeast Iran. Braz. J. Infect. Dis. 20, 379–383. doi: 10.1016/j.bjid.2016.05.003 27266592 PMC9427600

[ref35] Naser W. Maymand S. Dlugolenski D. Basheer F. Ward A. C. (2023). The role of cytokine-inducible SH2 domain-containing protein (CISH) in the regulation of basal and cytokine-mediated Myelopoiesis. Int. J. Mol. Sci. 24:12757. doi: 10.3390/ijms241612757 37628937 PMC10454631

[ref36] Naser W. Maymand S. Rivera L. R. Connor T. Liongue C. Smith C. M.. (2022). Cytokine-inducible SH2 domain containing protein contributes to regulation of adiposity, food intake, and glucose metabolism. FASEB J. 36:e22320. doi: 10.1096/fj.202101882R 35470501

[ref37] Nitcheu J. Bonduelle O. Combadiere C. Tefit M. Seilhean D. Mazier D.. (2003). Perforin-dependent brain-infiltrating cytotoxic Cd8+ T lymphocytes mediate experimental cerebral malaria pathogenesis. J. Immunol. 170, 2221–2228. doi: 10.4049/jimmunol.170.4.2221 12574396

[ref38] Palmer D. C. Guittard G. C. Franco Z. Crompton J. G. Eil R. L. Patel S. J.. (2015). CISH actively silences TCR signaling in Cd8+ T cells to maintain tumor tolerance. J. Exp. Med. 212, 2095–2113. doi: 10.1084/jem.20150304 26527801 PMC4647263

[ref39] Pongponratn E. Turner G. D. Day N. P. Phu N. H. Simpson J. A. Stepniewska K.. (2003). An ultrastructural study of the brain in fatal *Plasmodium falciparum* malaria. Am. J. Trop. Med. Hyg. 69, 345–359. doi: 10.4269/ajtmh.2003.69.345 14640492

[ref40] Sobah M. L. Liongue C. Ward A. C. (2021). Socs proteins in immunity, inflammatory diseases, and immune-related cancer. Front. Med. 8:727987. doi: 10.3389/fmed.2021.727987 PMC8481645 34604264

[ref41] Sun L. Jin Y. Q. Shen C. Qi H. Chu P. Yin Q. Q.. (2014). Genetic contribution of CISH promoter polymorphisms to susceptibility to tuberculosis in Chinese children. PLoS One 9:e92020. doi: 10.1371/journal.pone.0092020 24632804 PMC3954833

[ref42] Taylor W. R. J. Hanson J. Turner G. D. H. White N. J. Dondorp A. M. (2012). Respiratory manifestations of malaria. Chest 142, 492–505. doi: 10.1378/chest.11-2655 22871759

[ref43] Trengove M. C. Ward A. C. (2013). Socs proteins in development and disease. Am. J. Clin. Exp. Immunol. 2, 1–29. 23885323 PMC3714205

[ref44] Van Der Heyde H. C. Nolan J. Combes V. Gramaglia I. Grau G. E. (2006). A unified hypothesis for the genesis of cerebral malaria: sequestration, inflammation and hemostasis leading to microcirculatory dysfunction. Trends Parasitol. 22, 503–508. doi: 10.1016/j.pt.2006.09.002 16979941

[ref45] Wang Y. Wang W. (2010). CISH and susceptibility to infectious diseases. N. Engl. J. Med. 363:1676. doi: 10.1056/NEJMc1007642 20973148

[ref46] Weatherall D. J. Clegg J. B. (2002). Genetic variability in response to infection: malaria and after. Genes Immun. 3, 331–337. doi: 10.1038/sj.gene.6363878 12209359

[ref47] World Health Organization. (2022). World malaria report. Available at: https://www.who.int/teams/global-malaria-programme/reports/world-malaria-report-2022

[ref48] Yanez D. M. Manning D. D. Cooley A. J. Weidanz W. P. Van Der Heyde H. C. (1996). Participation of lymphocyte subpopulations in the pathogenesis of experimental murine cerebral malaria. J. Immunol. 157, 1620–1624. doi: 10.4049/jimmunol.157.4.1620 8759747

[ref49] Yang X. O. Zhang H. Kim B. S. Niu X. Peng J. Chen Y.. (2013). The signaling suppressor CIS controls proallergic T cell development and allergic airway inflammation. Nat. Immunol. 14, 732–740. doi: 10.1038/ni.2633 23727894 PMC4084713

[ref50] Yoshimura A. Naka T. Kubo M. (2007). Socs proteins, cytokine signalling and immune regulation. Nat. Rev. Immunol. 7, 454–465. doi: 10.1038/nri2093 17525754

[ref51] Yoshimura A. Ohkubo T. Kiguchi T. Jenkins N. A. Gilbert D. J. Copeland N. G.. (1995). A novel cytokine-inducible gene CIS encodes an SH2-containing protein that binds to tyrosine-phosphorylated interleukin 3 and erythropoietin receptors. EMBO J. 14, 2816–2826. doi: 10.1002/j.1460-2075.1995.tb07281.x 7796808 PMC398400

[ref52] Zhao L. Chu H. Xu X. Yue J. Li H. Wang M. (2014). Association between single-nucleotide polymorphism in CISH gene and susceptibility to tuberculosis in Chinese Han population. Cell Biochem. Biophys. 68, 529–534. doi: 10.1007/s12013-013-9733-2 23949851

