# Supplementary figures and images for: Cish knockout mice exhibit similar outcomes to malaria infection despite altered hematopoietic responses
Source: Front Microbiol. 2023 Nov 2;14:1288876. doi: 10.3389/fmicb.2023.1288876 (PMC10653303; doi:10.3389/fmicb.2023.1288876)

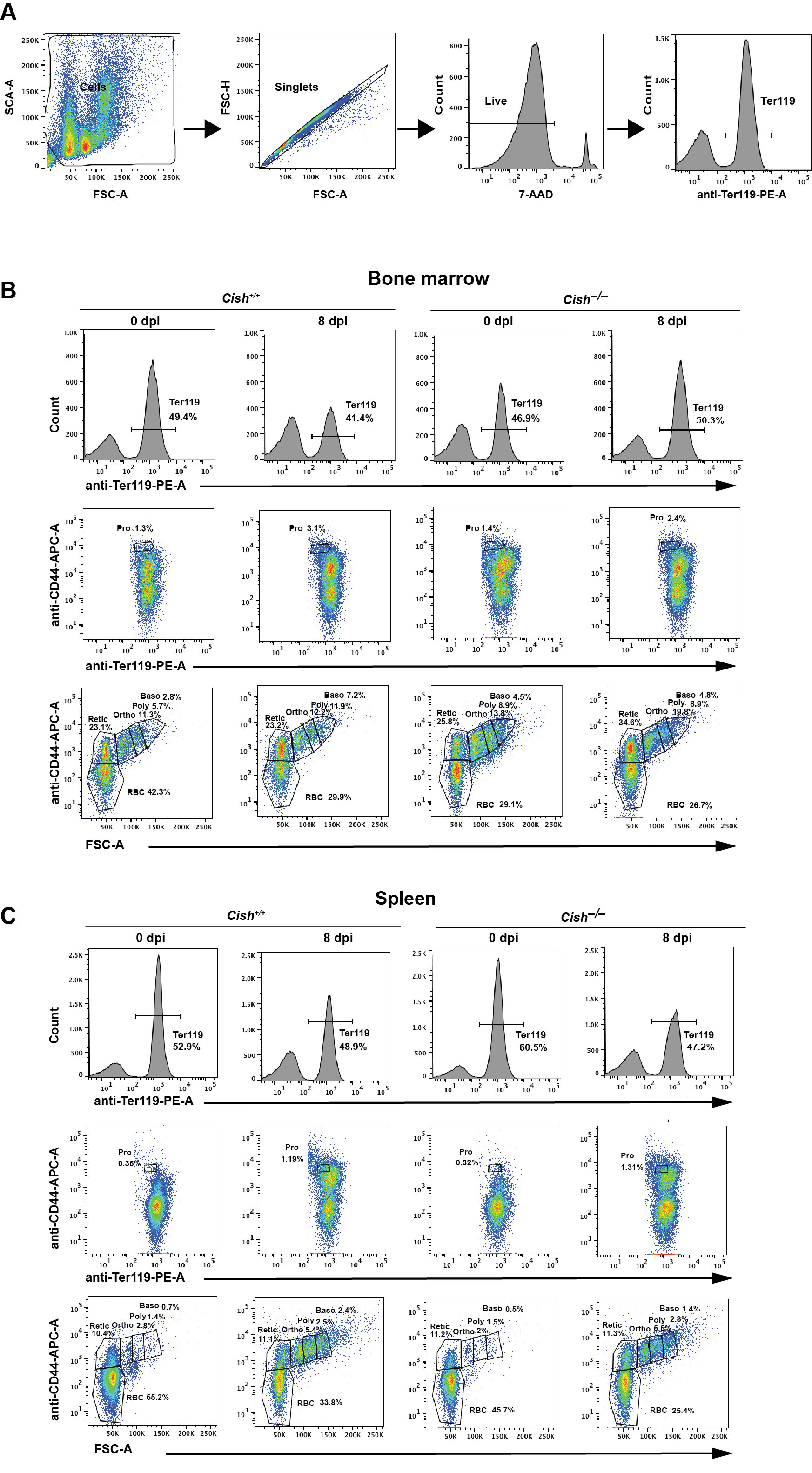

Supplement: Supplementary file 1 [file Image_1.JPEG]

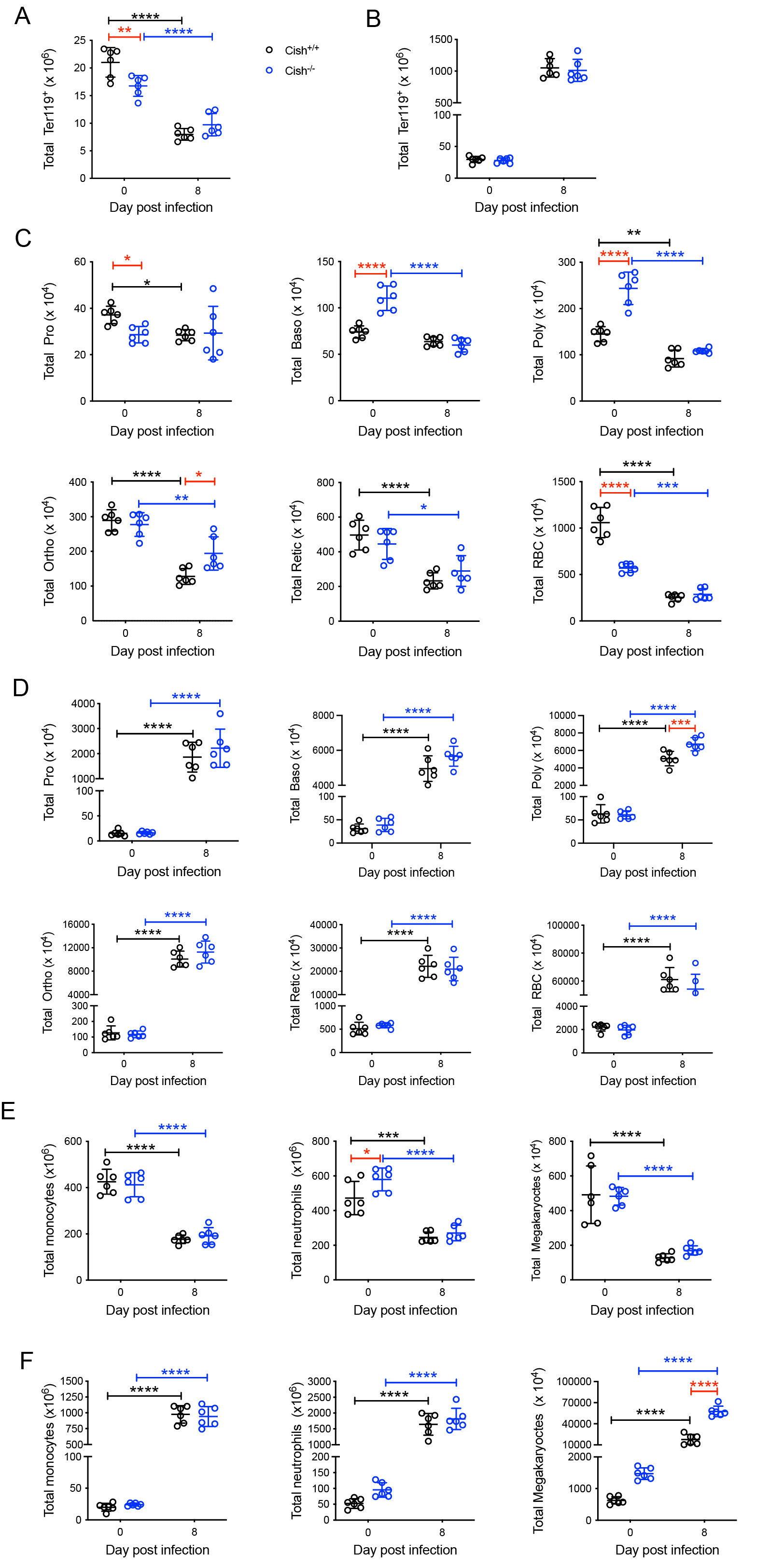

Supplement: Supplementary file 2 [file Image_2.JPEG]

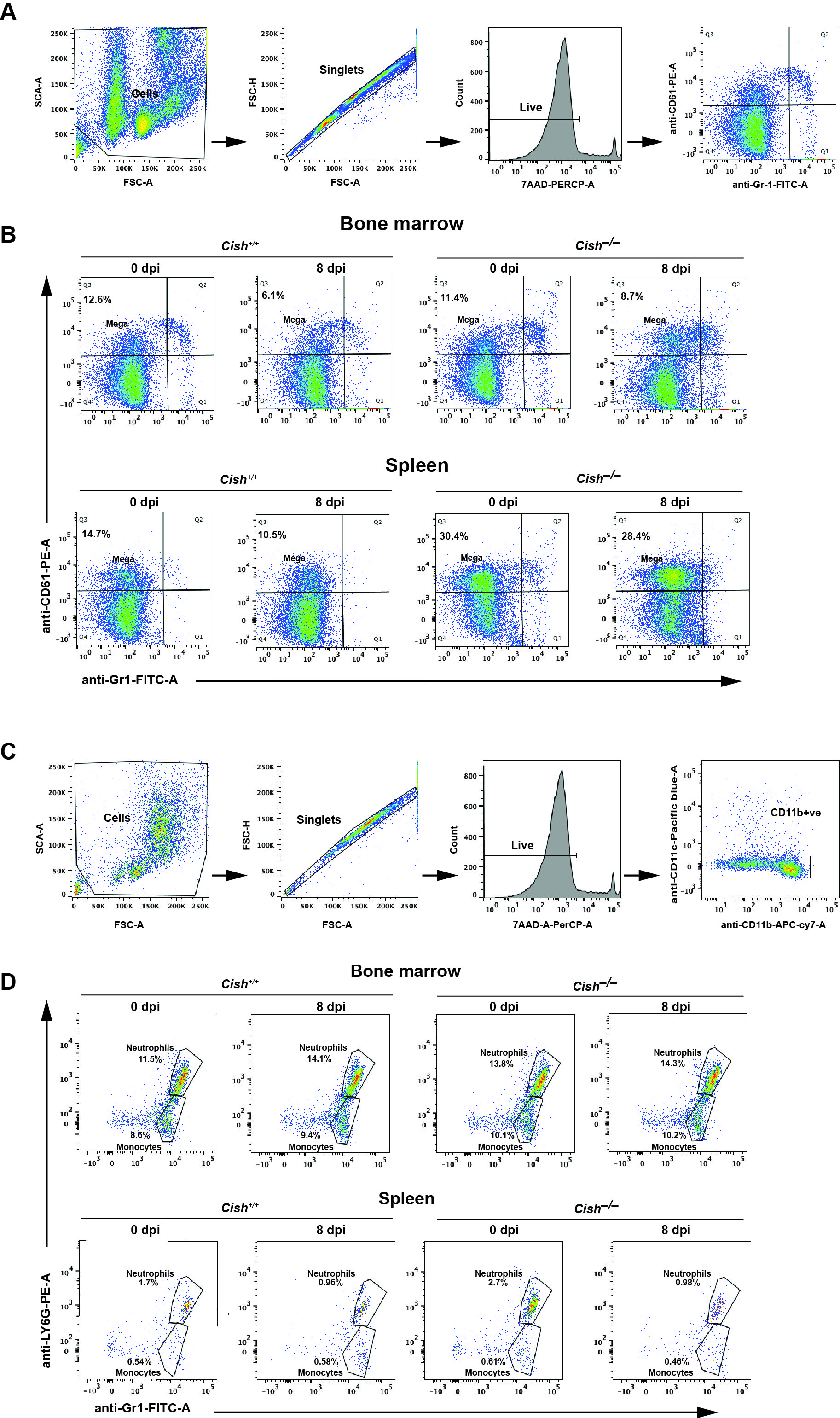

Supplement: Supplementary file 3 [file Image_3.JPEG]
